# The application of mesenchymal stromal cells (MSCs) and their derivative exosome in skin wound healing: a comprehensive review

**DOI:** 10.1186/s13287-021-02697-9

**Published:** 2022-01-24

**Authors:** Donghui Bian, Yan Wu, Guodong Song, Ramyar Azizi, Amir Zamani

**Affiliations:** 1 Department of Burns and Plastic Surgery, 960 Hospital of the People’s Liberation Army, Jinan, 250031 China; 2 Department of Burns and Plastic Surgery, Central Hospital Affiliated to Shandong First Medical University, Jinan, Shandong, 250013 China; 3 Department of Immunology, Medicine Faculty, Tabriz University of Medical Science, Tabriz, Iran; 4 Shiraz Transplant Center, Abu Ali Sina Hospital, Shiraz University of Medical Sciences, Shiraz, Iran

**Keywords:** Mesenchymal stromal cells (MSCs), Exosome, Wound healing, Differentiation, Paracrine factors

## Abstract

Recently, mesenchymal stromal cells (MSCs) and also their exosome has become a game-changing tool in the context of tissue engineering and regenerative medicine. MSCs due to their competencies to establish skin cells, such as fibroblast and keratinocyte, and also their unique attribute to suppress inflammation in wound site has attracted increasing attention among scholars. In addition, MSC’s other capabilities to induce angiogenesis as a result of secretion of pro-angiogenic factors accompanied with marked anti-fibrotic activities, which mainly mediated by the releases matrix metalloproteinase (MMPs), make them a rational and effective strategy to accelerate wound healing with a small scar. Since the chief healing properties of the MSCs depend on their paracrine effects, it appears that MSCs-derived exosomes also can be an alternative option to support wound healing and skin regeneration as an innovative cell-free approach. Such exosomes convey functional cargos (e.g., growth factor, cytokine, miRNA, etc.) from MSCs to target cells, thereby affecting the recipient skin cells’ biological events, such as migration, proliferation, and also secretion of ECM components (e.g., collagen). The main superiorities of exosome therapy over parental MSCs are the diminished risk of tumor formation and also lower immunogenicity. Herein, we deliver an overview of recent in vivo reports rendering the therapeutic benefits of the MSCs-based therapies to ease skin wound healing, and so improving quality of life among patients suffering from such conditions.

## Introduction

Wound healing denotes an important challenge in plastic surgery or other pathological conditions, such as diabetic foot ulcers (DFUs), epidermolysis bullosa (EB), etc. [1, 2]. Chronic wounds bring about marked patient morbidity, with unfavorable influences on patient quality of life, and also enhancing pain, stress as well as depression [3, 4]. Present standards of wound care emphasize recognizing and eliminating corresponding or aggravating factors with the anticipation of plummeting inflammation and subsequently progression of healing process [5]. Such therapeutic modalities are mainly expensive, time-consuming, and inefficient. Also, more than 50% of chronic wounds show substantial resistance to conventional treatments [6], and also these strategies cannot amend scarring [7].

Mesenchymal stromal cells (MSCs) have developed as an influential method to recover skin wound healing. In addition to the differential competence, MSC's other attributes such as ease of harvest, low immunogenicity accompanied with their ultimate role in native wound healing physiology introduce them a rational, safe, and also effective therapeutic approach [8, 9]. In fact, MSCs entice skin cell migration, angiogenesis, reepithelialization along with granulation tissue formation, leading to accelerated wound closure. Interestingly, they support a regenerative, but not fibrotic, wound healing milieu [10, 11]. Though clinical trials imply that MSC-based treatments are safe, feasible, and effective [12, 13], these trials are inadequate because of the restricted sample size and lacking long-standing follow-up. MSCs largely elicit their therapeutic influences by a diversity of actions on numerous cell types, and among all the wound healing phases, ranging from hemostasis to remodeling [14]. Exosomes derived from MSCs denote biological roles like the parental cells, and so can support tissue regeneration as a result of relocating their contents to surrounding cells [15].

Exosomes as a types of the extracellular vesicles (EVs) with a diameter in the range of 40–150 nm surround various bioactive molecules, comprising lipids, proteins, messenger RNA (mRNAs), transfer RNA (tRNA), long noncoding RNAs (lncRNAs), mitochondrial DNA (mtDNA), and also microRNAs (miRNAs) [16]. In addition to some general molecules, they also contain exclusive tissue type-specific proteins, reflecting their origins. At a measurable level, exosome secretion has strongly been validated from various cell types, such as immune cells [17–19], tumor cells [20, 21], and also MSCs [22–25]. MSC-derived exosomes comprise mRNAs, miRNAs, cytokines, and growth factors [26, 27]. Growing proof proposes that MSC-derived exosomes may exemplify an alternative cell-free approach with substantial benefits over parent MSCs, more importantly, compromised risk of tumor formation concomitant with lower immunogenicity [28].

In the current review, we have focused on the therapeutic merits of MSCs and also MSCs-derived exposure in skin wound healing and skin regeneration, with special emphasis on in vivo reports.

## The history of MSCs

The common notion of mesenchymal stem cells (MSCs), a term which was coined by Caplan in 1991, mainly relies on classical examination representing that transplantation of bone marrow (BM) to heterotopic anatomical sites led to de novo establishment of ectopic bone and marrow [29]. As a result of their marked differentiation capabilities into specialized cells developing from mesoderm, they were termed mesenchymal stromal cells (MSCs) [30]. Albeit, it was Friedenstein et al. who for the first time supposed that the osteogenic capacities, as demonstrated by heterotopic transplantation of BM cells, relied on a minor subpopulation of BM cells in the 1960s and 1970s [31, 32]. Based on their observations, rapid adherence to tissue culture vessels concomitantly fibroblast-like morphology of their progeny in culture makes them divergent from the common hematopoietic cells. Friedenstein et al. also found that cultivation of BM cell suspensions at clonal density might cause the formation of distinct colonies begun by single cells (the colony-forming unit fibroblastic, CFU-Fs) [31, 32]. They validated the clonal natures of each colony via various methods, in particular, the linear reliance of colony formation on the frequency of cells transplanted and also time-lapse photography [31, 32]. In 1978, the conception suggested by Schofield,which indicated that hematopoietic stem cells (HSCs) are adjusted by their physical interfaces with a particular cellular microenvironment within BM, outlined the importance of the existence of the another kind of stem cell (MSCs) in addition to HSCs on BM [33]. The idea of MSC was evolved from the historical origins of the conceptualized nonhematopoietic stem cell existing in BM [34]. Following the development of the conception of MSCs, the International Society for Cellular Therapy (ISCT) provided the minimum principles to define MSCs. Such criteria involve expressing plastic adherence attribute, showing CD73, D90, and CD105 but not CD14, CD34, CD45, and human leucocyte antigen-DR (HLA-DR) and finally being able to generate adipocyte, chondrocyte, and osteoblast in vitro [35, 36]. Upon isolation of the human MSCs isolation from BM tissue, which was first carried out by Pittenger and colleagues [37], cells showing MSCs minimal criteria have been efficiently isolated from adipose tissue, dental pulps, endometrium, placenta, synovial fluid, peripheral blood, skin,, umbilical cord, muscles, Wharton’s jelly, etc. [38, 39]. Nowadays, because of their self-renewal, multipotency, availability, immunoregulatory competencies as well as little ethical issues, MSCs therapy has resulted in a paradigm shift in the context of tissue engineering and regenerative medicine, such as wound healing and skin regeneration [40–42].

## Wound healing process

**Fig. 1:**
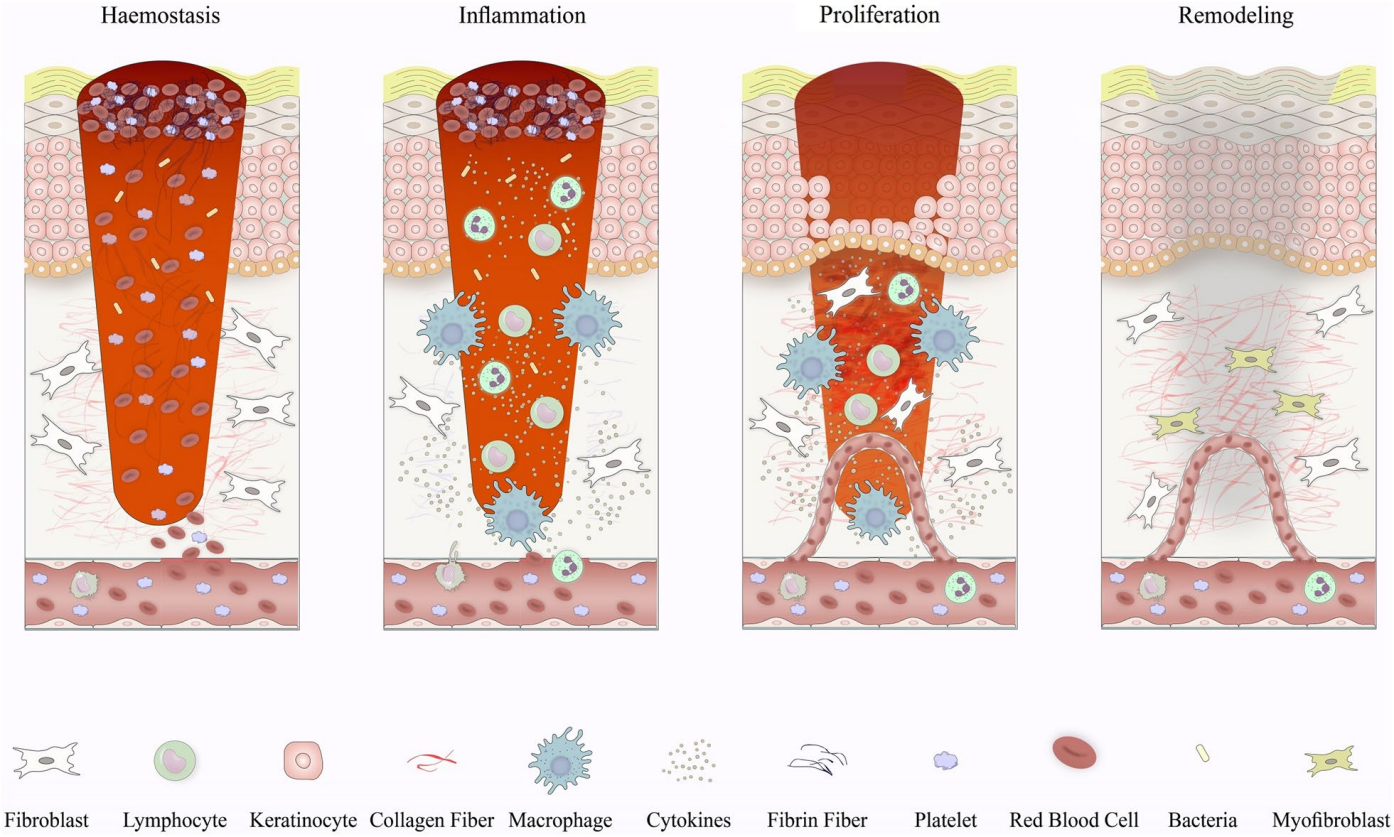
Schematic illustration of the phase and their responding cells involved in wound healing in vivo

Skin wound healing is a well-organized physiological process including the cooperation of various cell types as well as their derivative molecules [43, 44]. Cell and biochemical events during the process of wound repair are separated into the four main phases comprising the hemostasis phase, inflammatory phase, proliferative phase, and also remodeling (or maturation phase) (Fig. 1) [45]. Following the skin damage, hemostasis as the primary step of healing arouses immediately to end the bleeding. The interfaces between platelets with collagen give rise to platelets activation and thereby support their aggregation [46, 47]. Finally, platelet clumps change into a stable clot by thrombin, largely contributing to the generation of a fibrin mesh. The second phase of wound healing, the inflammatory phase, focuses on deteriorating bacteria and eradication of debris, offering the wound bed for the founding of novel tissue [48, 49]. Such events mainly depend on the activation of neutrophils and macrophages, which modify bleeding and also dampen infection via direct functions or the release of multiple soluble mediators [50]. The proliferative stage of wound repair comprises wound’s filling, wound’s margins contraction through myofibroblasts functions as well as the wound’s covering, named also epithelialization [51, 52]. Meanwhile, the creation of a new complex of blood vessels is urgently required to provide sufficient oxygen and nutrients to fresh granulation tissue [53]. In the remodeling phase, slow resolution of the inflammatory phase, collagen deposition, and covering of injured site wholly through the fresh tissues and lastly formation of scar tissue is succeeded [54]. Constructed tissue slowly attains strength and flexibility, and also collagen is remodeled from type III to type I, and the wound entirely closes [54].

## Exosome biogenesis

**Fig. 2:**
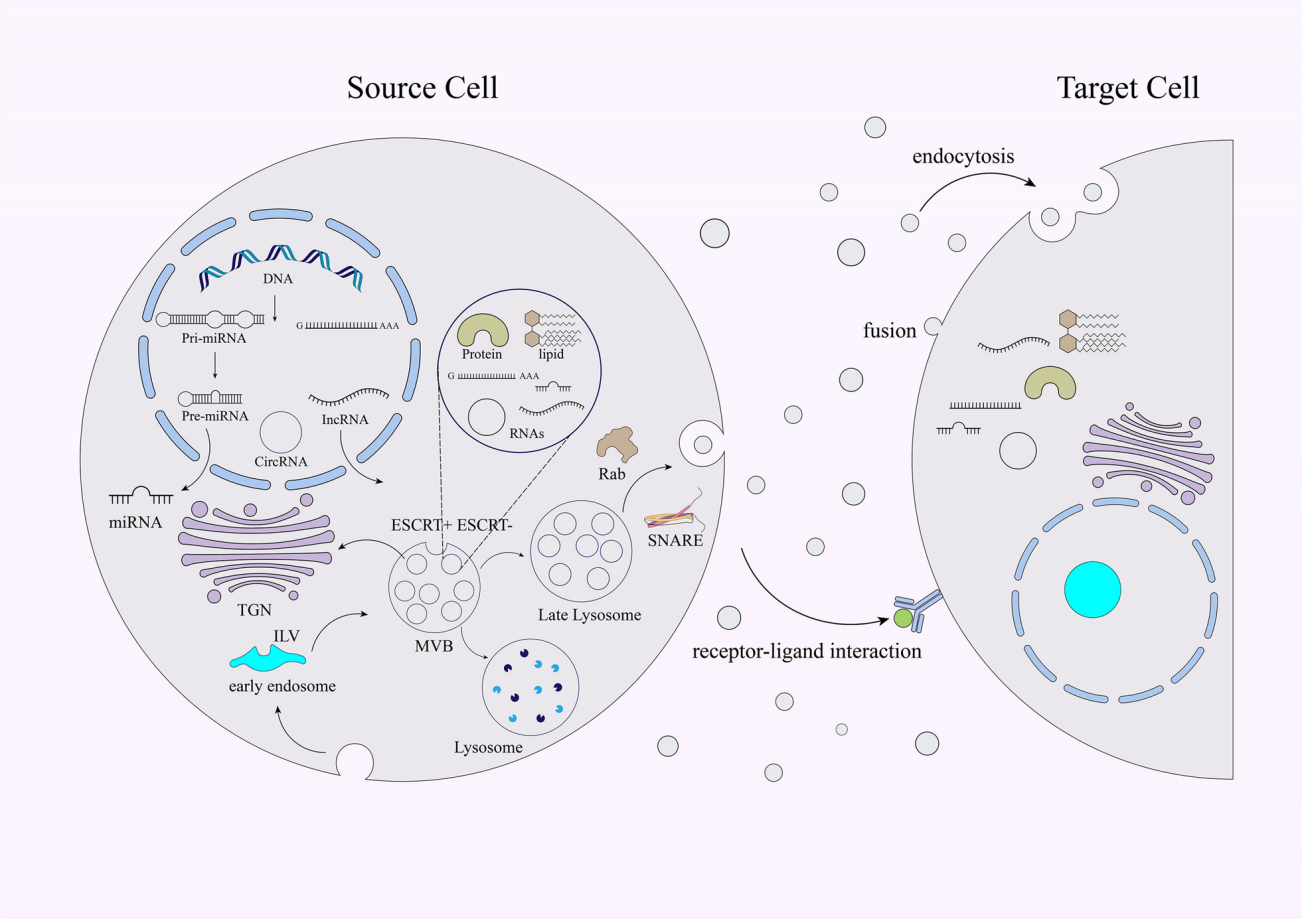
Schematic illustration of the biogenesis, compositions, and also release of the exosome. Following MVB incorporation with the cellular membrane, the release of exosome into the extracellular space is accomplished, and finally the released molecules are conveyed to recipient cells through endocytosis, or direct membrane fusion, or receptor‐ligand interfaces. Intraluminal vesicles (ILVs), Endosomal complexes required for transport (ESCRT), Multivesicular bodies (MVBs), Trans-Golgi network (TGN), Ras-related in the brain (Rab), Soluble NSF attachment protein receptor (SNARE)

Exosomes are one group of extracellular vesicles (EVs) with 40–150 nm in diameter. The first dependable study signifying the attendance of EVs was reported in 1946 by Chargaff and Wes [55]. In the 1980s, other studies conducted by Harding and Johnstone presented that transferrin receptors were released in correlation with small 50 nM vesicles from immature blood cells into the extracellular space by receptor-mediated endocytosis and recycling [56, 57]. In 1985, such vesicles were called exosomes by Johnstone [57]. The exosome biogenesis is a tightly adjusted procedure involving three chief steps; endocytic vesicle’s generation through invagination of the plasma membrane, formation of multivesicular bodies (MVBs) through inward budding of the endosomal membrane, and eventually shaping communication between generated MVBs and plasma membrane and release of the vesicular composites, termed exosomes (Fig. 2) [58, 59]. The exosome is comprised of lipid bilayer along with small cytosol lacking cellular organelles [59]. After secretion, exosomes perform as messengers, and so ease interfaces with recipient cells by the procedure of vesicular docking and incorporation. This event is facilitated by soluble N-ethylmaleimide-sensitive factor attachment protein receptor (SNAREs) complexes and also the endosomal sorting complex required for transport (ESCRT) [60–62]. Notwithstanding, MVB biogenesis can be completed without ESCRTs, as shown by the generation of intraluminal vesicles (ILVs) without the participation of ESCRT complexes [60, 61]. Further, lipids, such as ceramide [63], and also heat shock proteins (HSPs), lactadherin, GTPases, annexins, platelet-derived growth factor receptors (PDGFR), and tetraspanins involve in exosome biogenesis [64, 65]. As well, anterograde and retrograde protein-sorting steps between the Golgi and the plasma membrane is enabled by v-SNAREs and t-SNAREs [64]. Generally, the protein compositions of exosomes mainly depend on their origin cell [16].

## Exosome characterization

Assessment of the physicochemical possessions of exosomes, more importantly, size, shape, surface charge, density as well as porosity, is essential for determining their biological interactions. So, the correct determination of these attributes is of paramount significance. Various methods have been usually applied to characterize exosomes, such as biophysical, molecular, and microfluidic techniques [66]. Biophysical methods are used to characterize the exosomal size range. Such methods include nanoparticle tracking analysis (NTA), dynamic light scattering (DLS), tunable resistive pulse sensing (TRPS), flow cytometry (FACS), transmission electron microscopy (TEM), and atomic force microscopy (AFM) [64].

Biophysical methods are utilized to determine the size range of exosomes. NTA is one of the most critical biophysical approaches, determining the exosome concentration and size distribution in the 10 nm to 2 µm range [67]. These methods facilitate the tracking of the Brownian movement of NPs in a liquid suspension [68]. In fact, NTA estimates the exosome motion, which correlates with the particle size, through tracking each particle using the image analysis [67, 68]. The outputs of NTA involve the phenotype of the particles, size distribution, and also concentration. Apart from easy preparation, the valued merits of such techniques are detection of different EVs, such as exosomes, and estimating small particles with diameters of 30 nm [69].

DLS is another technique that measures the exosome size [70]. The passing of the monochromatic coherent laser beam by a particle suspension is the utmost basis of DLS [70]. Time-reliant variations in scattering intensity are inspired by constructive and destructive interference, which arise from particle’s relative Brownian motions within a suspension [70, 71]. Although DLS cannot visualize the particles, it can measure particles ranging in size from 1 nm to 6 µm. Further, it cannot provide any biochemical information rendering the cellular origin of EVs [72].

Recently, TRPS has appeared as another technique used in this context [73, 74]. The striking property of TRPS is the in situ single-particle characterizations and also exosome’s concentration quantity [75]. It is utilized to estimate a diversity of NPs suspensions, involving magnetic beads and a diversity of biomolecules. Notwithstanding, it has been noted that TRPS-based measurements are vulnerable to system stability problems [75].

AFM is a reliable technique and senses and simultaneously records interfaces between sample surface and probing tip [76, 77]. It is employed as a nanoscale mean to define the frequency, biomechanics, morphology, as well as biomolecular structure of exosome. This mean has the capability to estaimate samples in native circumstances with minimal provision [78, 79].

TEM is another tool widely utilized to characterize the morphology, structure, and size of several biological ingredients, such as exosomes [80]. The acting basis of TEM is the making of images following passing a beam of electrons by a sample, where a secondary electron is produced [81]. Such electrons are congregated and then magnified by particular lenses. in The sample’s fixation in glutaraldehyde and dehydrated is required, and also TEM images must be provided under a vacuum [82]. It is applied absolutely for the visualization of particles and the images achieved could be utilized for vesicular diameter quantities. However, the electron beam may harm biological specimens [83].

Another technique, flow cytometry or FACS, is a molecular mean utilized to evaluate exosomal surface proteins, and also offers the possibility to measure exosome size and structure [84, 85]. This tool is one of the most continually applied means for EV analysis, because it can define the cellular origin of solitary exosome. The primary specimen volume largely affects the both isolation and characterization of exosomes by FACS [86]. It supports the investigation of various physical and chemical features of cells and particles in suspension and also enables estimating the exosome both structure and also size. The acting principle of a FACS is that a laser beam with a particular wavelength is guided by a stream of fluid encompassing suspended particles previously labeled with fluorescent dyes [87]. The rapid quantification of suspended exosomes, detecting EVs smaller than 300 nm, and also classification and separation based on the levels of antigen expression are pivotal merits of FACS [88].

In addition to the cited techniques, some other molecular strategies, such as Raman spectroscopy are applicable to characterize exosomes [64]. Besides, a microfluidic-based mean has been utilized to determine the binding of exosomes to particular antibodies on microfluidics channels and consequently to bound vesicles elution. Exosomes also can be characterized by the existence of their load molecules (e.g., RNA), which can be evaluated using next-generation sequencing, microarray analysis, as well as digital droplet PCR [64].

## MSCs components complicated in wound healing

### Anti-inflammatory factors

MSCs can intensely target immune responses throughout tissue repair and offer a suitable soil for tissue repair and regeneration [89]. In addition to MSC's direct cell-to-cell contact, the mechanisms complicated in such immunomodulatory impacts are likely related to the capacity of MSCs to secret several soluble factors affecting the target cell biological process [90, 91]. In fact, MSCs modify the adaptive and innate immunological reactions by the abrogation of T cells activities, inhibition of the maturation of dendritic cells (DCs), plummeting B-cell activation and proliferation, and finally inhibiting NK cells activation and resultant cytolytic effect on target cells. Of course, inspiring T regulatory (Treg) cells proliferation and function by either paracrine effect or cell–cell contact mechanisms sustains MSCs mediated immunoregulation [92, 93]. Paracrine effect as shown is exerted by soluble mediators, including transforming growth factor-β1 (TGF-β1), prostaglandin E2 (PGE2), indoleamine-pyrrole 2, 3-dioxygenase (IDO), hepatocyte growth factor (HGF), nitric oxide (NO), and interleukin (IL-4), and IL-10 [92–94]. In damaged tissue, the existence of the pro-inflammatory cytokine, such as IFN-γ, tumor necrosis factor-α (TNF-α), IL-1α or IL-1β, may result in MSCs activation, and then support the release of various enzymes and soluble factors, counting IDO, PGE2 and cyclooxygenase 2 (COX-2) [92]. Such mediators potentiate MSCs mediated immunosuppressive functions. Meanwhile, PGE2 inhibits T-cell proliferation [95] and IDO affects the expansion as well as activation of immune cells by catalyzing the breakdown of tryptophan, which is required for T cell effector functions [96]. Further, NO produced by MSCs can hinder immune cell growth by suppressing signal transducer and activator of transcription 5 (STAT5) phosphorylation [97], and also through interfaces with various enzymes, ion channels, and also receptors [98]. Given that that suppression of inducible NO synthase (iNOS) generation in MSCs leads mainly to the improved T-cell activity, it seems that NO plays pivotal role in MSCs mediated immunoregulation [99] Also, MSCs therapy eases bacterial clearance through the augmenting migration and phagocytic functions of neutrophil by promoting IL-6, IL-8, and granulocyte–macrophage colony-stimulating factor (GM-CSF) levels. These molecules culminate removing of the infection and facilitate tissue repair as shown in wound healing [91].

### Proliferation-supportive factors

MSCs can secrete growth factors that can support the regeneration of tissues, and modifying MSCs to produce such growth factors can enhance their survival, proliferation, differentiation, and tissue reconstructing competencies [100]. Investigation of the potent influences of MSCs on target cell proliferation and growth factor expression has shown that MSC and their secretome can significantly enhance the proliferation rates of the target cell mainly by improving the expression of a spectrum of the growth factors, in particular, epidermal growth factor (EGF), and HGF [100]. These studies imply that MSCs improve the survival and proliferation of target cells chiefly in a paracrine manner [101].

HGF as one of the most central factors existing in MSCs-derived exosomes underlies positive regulation of varied cells proliferation by inducing HGF/cMET pathway [102]. For instance, it has previously been found that HGF significantly ameliorated reepithelialization in diabetic (DB) rats [103]. Moreover, HGF could stimulate the activities of matrix metalloproteinases type 2 and 9 (MMP-2 and MMP-9) participated in angiogenesis, cell migration, and also remodeling of ECM, supporting wound healing with the small scar in vivo [104]. Besides, EGF binding to EGF receptor (EGFR) is required for epidermal and hair follicle homeostasis, and also facilitates human keratinocyte stem cells proliferation in patients with extensive burns and genetic skin disorders. Meanwhile, a study revealed that that exosomes isolated from induced pluripotent stem cell (iPSC) derived MSCs (iMSCs) could promote growth of human keratinocytes (HaCaT) and human dermal fibroblasts (HDFs) by activating extracellular signal-regulated kinase (ERK)-1/2 signaling pathway [105]. These findings sugnified that iMSCs-exosome could stimulate the skin cells growth by inducing ERK1/2 possibly as a result of activating the EGFR-Ras-Raf axis [105]. Irrespective of ERK axes, ligand binding to EGFR can support autophosphorylation and resultant activation of both PI3K/AKT and STAT3 pathways, which in turn, stimulate skin cell proliferation as well as survival [106, 107].

### Pro-angiogenic factors

In addition to the direct differentiation, MSCs typically support angiogenesis by cell contact communication, and more importantly paracrine impacts. Pro-angiogenic factors secreted by MSCs involve bFGF, VEGF, placental growth factor (PGF), TGF-β, PDGF, angiopoietin-1, IL-6, and monocyte chemotactic protein-1 (MCP-1) [108]. Similarly, other studies have shown that MSCs trigger angiogenesis in vivo by secretion of VEGF, MCP-1, and IL-6 into their condition medium [109]. Importantly, such desired influences can be considerably compromised by pretreatment with neutralizing antibodies versus such molecules [109]. Meanwhile, IL-6 demonstrates proangiogenic, progrowth, and prosurvival attributes [110], and also MCP-1 acts as a chemoattractant for angiogenesis [111]. The most eminent proangiogenic factor, VEGF, is expressed by MSCs and also may stimulate MSCs differentiation [112]. VEGF adjusts endothelial cells' migrations and differentiation and also improves the endothelial cells recruitment for stimulating angiogenesis and endothelialization in wound tissue [113]. VEGFR2 stimulates the inducing multiple signaling axes, more importantly the stimulating mitogen-activated protein kinase (MAPK), PI3K/AKT, Src, and Rac axes [108]. These signaling axes, in turn, potentiate endothelial cells' survival, proliferation, and migration. Regardless of the cited factors, MSCs can also secrete IGF‑1, which reinforces endothelial progenitor cells proliferation [114]. It seems that binding IGF‑1 to IGF-1R on endothelial progenitor cells brings about activating PI3K/Akt signaling pathway and also supporting cell proliferation [114]. MSCs can also promote therapeutic angiogenesis in wound tissue and also treat serious vascular disorders by secreting HGF [115].

In addition to the cited competencies, MSCs can elicit skin cell differentiation to mature cells and also support anti-fibrotic attributes in wound tissue, ameliorating tissue repair [116, 117]. Further, they can increase the producing particular ECM components (e.g., collagen and fibronectin) in response to several damage-associated signals, as documented in the wound repair process [116, 118]. Vascular damages trigger disconnection of MSCs from the vascular wall. Then, such stem cells proliferate and secrete type 1 collagen and also fibronectin, engendering tissue repair after damage [118, 119].

## MSCs therapy in wound healing (preclinical studies)

### Direct application of MSCs

Human MSCs largely are known to proliferate and differentiate into skin cells to restore injured or dead cells, but also perform by an autocrine and paracrine pathway to stimulate cell regeneration and wound healing (Table 1). MSCs can migrate to wounded sites and differentiate into dermal fibroblasts (DF), endothelial cells, and keratinocytes. As well, MSCs and DFs can produce extracellular matrix (ECM) proteins complicated in supporting skin structure and activity. In sum, MSCs modify the macrophage's inflammatory phenotype contributed to the inflammatory phase, potentiate new blood vessels generation, leading to the promoting angiogenesis, and finally facilitate construction of granulation tissues, skin cells, and ECM production [120].

**Table 1:** MSCs therapy in animal models to accelerate wound healing and induce skin regeneration

Condition	Models	Treatment	Results	References
Excisional wound	Mice	BM-MSCs	Improved wound closure and collagen fibers	[121]
Excisional wound	Mice	BM-MSCs	Ameliorated wound closure along with increased re-epithelialization, cellularity, as well as angiogenesis	[122]
Leukocyte adhesion deficiency 1(LAD1)	Mice	BM-MSCs	Robust myofibroblast differentiation wound contraction, and also the development of vessel formation	[123]
Excisional wound	Mice	UCB-MSCs	Wound healing by MSCs differentiation into keratinocyte in the wound tissue	[124]
Excisional wound	Rat	MSCs co-cultured with SGCs	Recruitment to wound site and wound healing of skin appendages	[125]
Severe burn	Rat	UC-MSCs	MSCs migration into the wound and remarkably reduction in immune cell recruitment to the wound site, with reduced levels of IL-1, IL-6, TNF-α and promoted levels of IL-10 and TSG-6 in wounds	[126]
Excisional wound	Mice	UCB-MSCs	Fail to show positive effects on wound healing and reducing collagen deposition	[127]
		WJ-MSCs		
Excisional wound	Mice	WJ-MSCs	Potentiating the normal skin fibroblast proliferation	[128]
Excisional wound	Sheep	PB-MSCs	Fail to promote granulation tissue formation, neovascularization, structural proteins, and skin adnexa	[129]
Excisional wound	Mice	BM-MSCs	Low immunogenicity than fibroblast in vivo	[41]
Excisional wound	Mice	IL-1 primed gingiva-MSCs	Enhancing cell migration, and dermal–epidermal junction formation, supporting wound healing	[130]
Atopic dermatitis (AD)	Mice	Poly I:C or IFN-γ primed WJ-MSCs	Attenuation of epidermal thickness as well as inflammatory cell infiltration in skin lesions	[131]
Excisional wound	Rat	MSCs seeded on the artificial dermal matrix (ADM)	Inducing skin regeneration by reduced collagen deposition, promoted reepithelization, and neo-angiogenesis	[132]
Diabetic foot ulcers (DFU)	Rat	BM-MSCs seeded on biocompatible hydrogel	Suppression of M1 macrophages activities, promoting the M2 macrophages activities, inducing the granulation tissue formation and also angiogenesis	[133]
Excisional wound	Mice	BM-MSCs seeded on small intestinal submucosa (SIS)	Inhibition of inflammation of the wound and also increasing the skin regeneration-related growth factors in the wound site	[134]
Excisional wound	Mice	AT-MSCs seeded on ppAAc	Suppression of TNF-α-dependent inflammation, improvement of anti-inflammatory M2 macrophage numbers, and also triggering TGF-β1-mediated angiogenesis, myofibroblast differentiation, and finally the formation of granulation tissue	[135]
Diabetic foot ulcers (DFU)	Mice	MSCs seeded on PEG-based collagen hybrid scaffolds	Inducing angiogenesis leading to tissue repair	[136]
Excisional wound	Porcine	MSCs seeded on collagen	Improving MSC adhesion and infiltration and supporting the wound healing	[137]
Excisional wound	Mice	BM-MSCs seeded on ADM	Supported angiogenesis as well as collagen fiber structural remodeling	[138]
Excisional wound	Mice	MSCs seeded on fibrin hydrogels	Stimulating endothelial cell proliferation, promoting macrophage polarization log with improving angiogenesis	[139]
Excisional wound	Mice	ISCs and MSCs coencapsulated into a synthetic hydrogel	Higher healing response than singly delivered MSCs or ISCs	[140]

Mesenchymal stromal cells (MSCs), Adipose tissue (AT), Bone marrow (BM), Umbilical cord blood (UCB), Wharton's jelly (WJ), Sweat gland cells (SGCs), Insulin secreting cells (ISCs), Tumour necrosis factor α (TNFα), Transforming growth factor-beta (TGF-β), Insulin secreting cells (ISCs), Interleukin (IL), Polyethylene glycol (PEG), TNFα-stimulated gene-6 (TSG6), Plasma polymerisation with a thin layer of acrylic acid (ppAAc), Interferon-gamma (IFN-γ)

In vivo, studies have indicated that allogeneic BM-MSCs therapy caused substantial changes in the wound repair kinetics of lesions in the excisional wound splinting mice model. Murine treated with allogeneic MSCs experienced improved wound closure, and accelerated granulation tissue formation accompanied with less inflammatory response. Such desired events are most probably caused by MSCs secreted molecules including IGF-1, VEGF, MMP-1, keratinocyte growth factor (KGF), HGF, angiopoietin-2 (ANG-2), type 1 collagen (COL1), and PGE2 [121]. BM-MSCs, but not CD34 + BM cells in the wound, showed the keratinocyte-specific protein keratin and shaped glandular structures, presenting a straight involvement of stem cells in skin recovery in mice models [122]. Wu et al. also displayed that MSCs could produce high levels of VEGF and angiopoietin-1, indicating that stem cells ameliorate wound healing by differentiation and also secretion of angiogenesis-inducing molecules [122]. Other reports also have exhibited that MSCs by secreting different cytokines and chemokines, including angiopoietin-1 (ANG-1), stromal-derived factor-1 (SDF-1), VEGF-α, IGF-1, EGF, KGF, macrophage inflammatory protein (MIP)-1 alpha and beta and also erythropoietin support normal wound healing [141]. It seems that regardless of the other cited mechanisms, promoting the migration of macrophages and keratinocytes underlies wound repair in vivo [141]. Besides, administration of AT-MSCs could sustain normal healing of CD18 − / − wounds by recovering the reduced TGF-β1 levels in leukocyte adhesion deficiency syndrome type 1 (LAD1) murine model [123]. TGF-β1 secreted from MSCs could bring about improved myofibroblast differentiation, wound contraction, and concomitantly vessel creation [123]. Importantly, low TGF-β1 concentrations as shown in CD18 − / − wounds may stimulate TGF-β1 secretion from MSCs, while high TGF-β1 levels may inhibit TGF-β1 generation [123]. Moreover, local administration of UCB-MSCs labeled with 5-bromodeoxyuridine (BrdU) enhanced the healing of murine skin defect wounds by direct differentiation into keratinocytes in the wound site [124]. In another study, following co-culture with heat-shocked confluent sweat gland cells (SGCs), BrdU-labeled MSCs differentiated into SGCs and support sebaceous glands, hair follicles, blood vessels, and dermis in full-thickness wounds [125]. Following wound healing, a subset of labeled MSCs returned to the BM, while other was recollected in the dermis. These studies exposed that MSCs could dock at particular regions to ameliorate wound healing of skin appendages, and also to home toward marrow [125]. Also, in sheep skin, wounds treated with PB-MSCs experienced more appropriate cutaneous adnexa than the untreated wounds without any signs of inflammation or leukocytes [129]. As well, collagen1 gene (Col1α1) expression was also improved in the wound tissue in mice treated with MSCs than the control group. Hence, it was presented that MSCs did not stimulate an inflammatory reaction and also could ameliorate the attendance of granulation tissue, neovascularization, structural proteins, and skin adnexa in large animal models [129]. UC-MSCs also stimulate beneficial effects in severe burns rat models. Correspondingly, GFP labeled hUC-MSCs injection by intravenous route resulted in wound healing in vivo upon migration into wound tissue and attenuating the concentration of infiltrated inflammatory cells and levels of TNF-α IL-1 and IL-6, concomitant with improved levels of tumor necrosis factor- (TNF) stimulated gene-6 (TSG-6) and IL-10 in wounds [126]. Moreover, the higher ratio of collagen types I and III in the hUC-MSC treated group than the burn group [126]. Furthermore, a recent study revealed that UCB-MSCs expressed lower rates of the IL1A and IL1B, well-known pro-inflammatory cytokines, but improved rates of the ECM-degradation enzymes MMP1 and urokinase plasminogen activator (PLAU) than WJ-MSCs, representing that UCB-MSCs are more favorable stem cell source favor scarless wound healing [127]. Unfortunately, direct injection of neither UCB-MSCs nor WJ-MSCs into full-thickness skin defect sites did not support wound healing in nude mice, according to Doi et al. reports [127]. Another report also suggested that allogeneic fibroblasts were chiefly confined to the injection area, whereas allogeneic BM-MSCs migrated into the entire wound [41]. Also, allogeneic fibroblast, but not allogeneic BM-MSC, administration caused increased CD45 + leukocytes, CD3 + lymphocytes, and CD8 + T cells in wound tissue, demonstrating the lower allogenicity of MSCs than fibroblast in vivo [41].

### Primed-MSCs application

There is also clear evidence indicating that MSCs pretreatment with inflammatory cytokines, such as IL-1β [130], IFN-γ [131], and TNF-ɑ [142] may provoke wound healing aptitudes. For instance, IL-1β-primed gingival MSCs enhanced cell migration and dermal–epidermal junction generation, and also prohibited inflammation in vitro. Moreover, such cells ameliorated epidermal substitute engraftment in vivo [130]. Notably, IL-1β-primed gingival MSCs showed dissimilar secretory profiles from naive gingival MSCs, as shown by overexpression of TGF-β and MMP pathway agonists. These findings suggest that MMP-1, MMP-9, and TGF-β1 likely contribute to IL-1β-primed gingival MSC-elicited activities [130]. WJ-MSCs primed with the toll-like receptor 3 (TLR3) agonist poly I:C or interferon-γ (IFN-γ) also showed superiority over naïve cells in terms of the improved immunomodulatory activities, making primed WJ-MSCs a rational plan to sustain skin regeneration [131]. Likewise, TNF-α primed MSCs showed superiority over naïve cells to migrate into the wound tissue, and so underlie efficient wound healing as a result of improved expression of intercellular adhesion molecule-1 (ICAM-1) and vascular cell adhesion molecule-1 (VCAM-1) [142]. As well, the p38 signaling pathway might be participating in the TNF-α-induced migration capability of MSCs for wound repair and regeneration [142].

### MSCs-based delivery by biomaterials or other modalities

MSCs seeded on scaffolds have recently been used in regenerative medicine of skin to offer a more desired would heal effect in vivo. Compared to administration of cells alone, transplantation of MSCs in a biomaterial can increase their wound healing capability by localizing cells at the defect site and up-regulating trophic factor secretion. Irrespective of trophic factors, MSC-seeded scaffolds could up-regulate MMP9 expression in the ECM and also increase the recruitment of endogenous progenitors throughout tissue repair [132]. MSCs seeded on scaffold could greatly ameliorate the quality of regenerated skin, attenuate collagen deposition, enhance reepithelization and neo-angiogenesis, and ultimately sustain a greater return of hair follicles and SGs mainly by releases of paracrine factors [132]. Likewise, administration of MSC-seeded small intestinal submucosa (SIS) complex led to the successful migration of 27.6% of the MSCs into the wound site at the first attempt. Notably, recurrent transplantation of additional MSCs did not modify the number of MSCs contributing to wound healing [132]. This therapeutic strategy brought about inhibition of inflammation in the wound site along with enhancing the skin regeneration-related growth factors at the optimal number of about 3 × 10^5^ MSCs injected into the wound area [132]. Another report also has signified that medical-grade silicone-coated by plasma polymerisation with a thin layer of acrylic acid (ppAAc) as a carrier for AT-MSCs could potentate their capacities to accelerate wound healing in vivo most likely by suppressing TNF-α-dependent inflammation, intensifying M2 macrophage population, stimulating TGF-β1-mediated angiogenesis, myofibroblast differentiation as well as the establishing granulation tissue [132]. Based on the previous reports, acrylic acid hydrogel could enhance skin regeneration as evidenced in the burn wound murine model [143]. Thus, it seems that this intervention is rational and also operational in this context. In another study, E7 peptide, a peptide with a specific affinity for MSCs, was immobilized to a collagen scaffold by a collagen-binding domain (CBD) to establish a functional collagen scaffold [144]. In a porcine model, the investigation revealed that CBD-MSC-peptide collagen scaffold improved MSC competencies and also accelerated wound healing [144]. Thereby, it was evidenced that such scaffolds could augment the capable presence of MSCs in wound sites to serve better therapeutic outcomes. Recently, transplantation of GFP-MSCs-seeded on an acellular dermal matrix (ADM) scaffolds into surgical skin wounds in vivo demonstrated that injected cells were retained inside the regenerating skin till 2 weeks upon transplantation [138]. Given the observations, injected MSCs differentiated into functional cells and enabled recruitment of more endogenous cells for tissue remodeling by secreting soluble mediators. Notwithstanding, further analysis indicated that endogenous cells, but not exogenous cells, convinced skin wound healing during the later stage [138]. Also, MSC spheroids in stiffer gels were found that could produce higher levels of pro-angiogenic factor VEGF and anti-inflammatory factor PGE2 than naive MSCs, stimulating endothelial cell proliferation, improving macrophage polarization, and accelerating angiogenesis in vivo [139].

## MSCs-exosome in wound healing (preclinical studies)

Exosomes are one of the chief secretory yields of MSCs, resembling the influences of parental MSCs. As a cell-free approach, they can convey several proteins, mRNA, and miRNAs to modify the functions of recipient cells, and can facilitate skin wound healing. Exosomes are more convenient than MSCs to store and transfer biological molecules, and also compromise the risks associated with the cell application, providing a more safe and efficient therapeutic plan. Recently, it has been documented that MSCs-derived exosomes could induce wound healing in vivo (Table 2). The MSCs derived from various sources, such as BM, AT and UC contain remarkable levels of VEGF-A, FGF-2, HGF, and platelet-derived growth factor-BB (PDGF-BB). However, UC-MSCs include higher levels of TGF-β than MSCs derived from BM or AT [145]. All exosomes secreted by theses stem cells stimulated keratinocyte and fibroblast proliferation and migration. Of course, the impacts of exosomes on cell biological events have a relationship with exosome origins as well as their target cells [145]. For example, the more prominent induction of primary dermal fibroblasts belongs to exosomes derived from BM-MSCs [145]. As well, BM-MSCs- derived exosome inspired accelerated re-epithelialization, with an enriched expression of cytokeratin 19 (CK19), proliferating cell nuclear antigen (PCNA), collagen I in vivo [146]. These exosomes also improved proliferation and conversely plummeted apoptosis of skin cells following heat-stress in vitro. Such desired effects were likely resulted from the existence of Wnt4 in these exosomes, which could potentiate β-catenin nuclear translocation and functions to boost proliferation and migration of skin cells [146]. Furthermore, the induction of the AKT pathway by BM-MSCs-exosomes diminished the heat stress-induced apoptosis in a rat skin burn model [146]. UC-MSCs-exosomes also affected dermal fibroblasts-myofibroblasts transition via targeting the TGF-β1/Smad2/3 signaling pathway in vivo [147]. In damaged skin, fibroblasts are activated and differentiate into myofibroblasts, minimizing wound size and secreting ECM proteins.Similarly, UC-MSCs exosome enriched in specific microRNAs (miR-21, -23a, -125b, and -145) mediated anti-scarring effects of UC-MSCs both in vitro and in vivo by suppressing the TGF-β2/SMAD2 pathway, making them an effective source to avert scar formation during wound healing [148, 149]. Moreover, conditioned medium (CM) derived from amniotic fluid (AF)-MSCs containing VEGF and TGF-β1 improved the proliferation and migration of human dermal fibroblasts in vitro, and also augmented wound closure in vivo [150]. As well, hypoxia primed AF-MSC derived CM showed superiority over normal AF-MSC-CM, and accelerated wound healing by the enhancement of hypoxia-induced paracrine factors upon activation of TGF-β/SMAD2 and PI3K/AKT pathways [150]. Activation of such axes in skin resident cells ameliorates wound healing as a result of enhancing these cells' biological activities, such as proliferation and ECM secretion. Likewise, AT-MSC-derived CM therapy could reverse the initial stages of diabetic polyneuropathy (DPN), ducking the risk of lower limb amputation induced by foot ulcer and eventually provoking the wound healing procedure in diabetic mice [151]. Besides, in radiation-induced skin wound rat model, WJ-MSCs exosome by paracrine effects improved human umbilical vein endothelial cells (HUVECs) proliferation, recovery of sebaceous glands, and also angiogenesis with a small scar formation [152]. BM-MSCs derived CM also efficiently attenuated UV-induced MMP1 expression and sustained pro-collagen synthesis, which in turn, led to the amelioration of UV-induced skin damage in mice [153]. UC-MSCs-exosome also could promote the proliferation and also migration of human keratinocytes (HaCa) through the suppressing nuclear translocation of apoptosis-inducing factor (AIF) and also promoting poly ADP ribose polymerase 1 (PARP-1) expression in vitro [154]. As well, UC-MSCs-exosome ameliorated full-thickness skin wounds by improving epidermal re-epithelialization and dermal angiogenesis [154]. In addition, the beneficial impacts of the MSCs-exosome have recently been validated in animal models with systemic sclerosis (SSc), a rare disease characterized by the development of fibrosis in the skin [155]. It appears that specific miRNAs, such as miR-196b-5p and miR-29a-3p play key roles in this regard [155, 156].

**Table 2:** Application of MSCs-EVs (e.g., exosome) in animal models to accelerate wound healing and induce skin regeneration

Condition	Models	Cell Source	Results	References
Skin burn	Rat	BM	Accelerated re-epithelialization, and also augmented expression of CK19, PCNA, and collagen I in vivo	[146]
Excisional wound	In vitro	UC	Inhibition of fibroblasts-myofibroblasts transition by suppressing TGF-β1/Smad2/3 signaling axis	[147]
Excisional wound	Mice	UC	Inhibition of myofibroblast formation by suppressing the TGF-β2/SMAD2 axis via exosome enriched in miR-21, -23a, -125b, and -145	[148, 149]
Excisional wound	Mice	AF	Improving the proliferation and migration of human dermal fibroblasts, causing promoted wound closure by CM enriched in VEGF and TGF-β1	[150]
Diabetic foot ulcers (DFU)	Mice	AT	Amelioration of the wound healing procedure	[151]
Radiation dermatitis	Rat	WJ	Sustained HUVEC proliferation, restoration of sebaceous glands concomitant with small scar formation	[152]
Radiation dermatitis	Mice	BM	Promotion of MMP1 expression and inducing pro-collagen synthesis	[153]
Excisional wound	Mice	UC	Wound healing by intensifying the epidermal re-epithelialization and dermal angiogenesis	[154]
cGVHD	Mice	BM	Inhibition of the activity of IL-17-expressing Th17 and induction of IL-10-expressing Tregs	
Systemic sclerosis	Mice	BM	Marked anti-fibrotic influences of exosome enriched in miR-196b-5p	[155]
Excisional wound	Mice	BM	Amelioration of the scar pathological injury, reducing the inflammatory response and also attenuation of collagen deposition by TSG-6 overexpressed MSC-exosomes	[157]
Excisional wound	Rat	UC	Enhanced endothelial cell proliferation, migration, and angiogenic tubule formation with reduced scar formation by administration of exosome plus nanoparticles	[158]
Excisional wound	Mice	UC	Stimulated in vivo angiogenesis by exosome enriched in miR-135b-5p, and miR-499a-3p more evidently upon blue light illumination	[159]
Diabetic foot ulcers (DFU)	Rat	BM	Inducing the PI3K/AKT signaling pathway by miR-126 mediated PTEN downregulation, supporting angiogenesis	[160]
Excisional wound	Rat	UC	Wounds with faster and better resolution in three-dimensional culture-derived conditioned medium (CM3D) -treated wounds than two-dimensional culture-derived conditioned medium (CM2D) -treated wounds	[161]

Mesenchymal stromal cells (MSCs), Adipose tissue (AT), Bone marrow (BM), Umbilical cord (UC), Amniotic fluid (AF), Wharton's jelly (WJ), Tumour necrosis factor α (TNFα), Transforming growth factor-beta (TGF-β), TNFα-stimulated gene-6 (TSG6), Matrix metallopeptidases (MMPs), Proliferation cell nuclear antigen (PCNA), Cell keratin 19 (CK19), Human umbilical vein endothelial cells (HUVECs), Vascular endothelial growth factor (VEGF), Conditioned medium (CM). Extracellular vesicles (EVs), Phosphatase and tensin homolog (PTEN), Phosphoinositide 3-kinases (PI3Ks), Interleukin (IL), MicroRNAs (miRNAs), Regulatory T cells (Tregs), T helper 17 cells (Th17)

Also, some reports have signified that modification of parental MSCs or pretreatment or exposure with some ingredients could enhance the therapeutic effects of MSCs-exosome to support more favorable wound healing [157, 159, 160]. For example, subcutaneous transplantation of TSG-6 overexpressed MSC-derived exosomes effectively altered scar pathological injury, reduced inflammatory molecular production, and also decreased collagen deposition in a mouse skin wound model [157]. As well, iron oxide nanoparticle (NP)-labeled MSCs-exosomes showed superiority in terms of inducing endothelial cell proliferation, migration, and angiogenic tubule formation in vivo over NP-treated MSCs [158]. The NP-labeled MSCs-exosomes also attenuated scar formation and improved CK19, PCNA, and collagen expression in vivo, indicating the feasibility of such modalities in skin wound repair [158]. Moreover, investigation of in vivo angiogenic capacity of blue light-treated MSC-derived exosomes using both murine matrigel plug and also skin wound models exhibited that blue (455 nm) light illumination could enhance the therapeutic impacts of MSC exosomes by increasing their proangiogenic potential in vitro and in vivo via the promotion of the miR-135b-5p and miR-499a-3p [159]. It has previously been displayed that these miRNAs are largely involved in regulating wound healing, and inflammation, and also could ameliorate wound healing [159]. On the other hand, exosomes derived from BM-MSCs preconditioned by deferoxamine exhibited more evident proangiogenic potential in wound repair [160]. These exosomes improved vein HUVECs proliferation in vitro by activating the PI3K/AKT signaling pathway through miR-126 mediated downregulation of the phosphatase and tensin homolog (PTEN) [160]. Moreover, they accelerated wound healing and angiogenesis in streptozotocin-induced diabetic rats [160].

## MSCs and their exosomes in wound healing and skin regeneration (clinical trials)

Several trials have been carried out or are ongoing to address the safety, feasibility, and efficacy of MSCs-based therapeutics to serve wound healing and skin regeneration in the human subject (Table 3). Although some reports have indicated that UC-MSCs could not ameliorate skin regeneration for instance in cesarean section skin scars [162], various clinical trials have evidenced MSC's safety and efficacy in this regard [12, 163, 164].

**Table 3:** The clinical trials rendering MSCs-based therapy to accelerate wound healing and induce skin regeneration

Condition	Cell source	Results	References
Cesarean section skin scars	UC	Fail to facilitate skin regeneration in cesarean section skin scars	[162]
Ablative fractional laser (AFL)	AT	Skin regeneration by reduced MMP-1 and MMP-2 expression and also promoted collagen 1 expression	[165]
Ablative fractional laser (AFL)	UC	Ameliorated wound healing and reduced post-treatment erythema by MSCs containing serum and cream	[166]
Diabetic foot ulcers (DFU)	UC	Supporting greater and more stable wound repair	[167]
Diabetic foot ulcers (DFU)	WJ	Verification of the safety and efficacy of acellular amniotic membrane seeded with WJ-SCs with a robust reduction in wound size	[168]
Diabetic foot ulcers (DFU)	BM	Attenuated wound size and also improved vascularity of the dermis by combination therapy with fibroblasts on biodegradable collagen membrane accompanied with autologous BM-MSCs	[169]
Bullosis diabeticorum (BD)	BM	Ameliorated clinical outcomes and prohibited lower limb amputation	[170]
Epidermolysis bullosa (EB)	UC	Stimulation of M2 macrophage polarization and reducing mast cell infiltration in EB skin leading to diminished pain score and wound healing	[171]
Chronic plantar ulcers in leprosy (CPUL)	AM	Stimulated wound healing by administration of MSCs-CM plus vitamins C or E	[163]

Mesenchymal stromal cells (MSCs), Adipose tissue (AT), Bone marrow (BM), Umbilical cord (UC), Amniotic membrane (AM), Wharton's jelly (WJ), Matrix metallopeptidases (MMPs), Conditioned medium (CM)

Recently, a study on 24 patients with aging skin who underwent ablative fractional laser (AFL) treatment revealed that AT-MSCS-CM induced beneficial effects most likely by decreased MMP-1 and MMP-2 expression along with improved collagen 1 expression [165]. Likewise, administration of allogeneic UC-MSCs containing serum and cream instigated accelerated wound healing and diminished post-treatment erythema, and thereby exhibited the significant competence to support recovery after AFL [166]. Evaluation of the possible therapeutic merits of UC-MSCs transplantation in 53 patients (72 limbs) with DFU also exhibited that the intervention group experienced considerably greater and more stable amelioration in ankle-brachial pressure index, transcutaneous oxygen tension, skin temperature, and claudication distance compared to control group [167]. Further, robust enhancement in neovessels concomitant with complete or gradual ulcer healing was detected in the experimental group without any drastic complications or untoward treatment-related reactions [167]. Also, a randomized clinical trial in 5 patients with chronic diabetic wounds verified the safety and efficacy of acellular amniotic membrane seeded with WJ-SCs during 1-month follow-up, as shown by significant alteration in the percentage and period of wound healing and the size of the wound [168]. After 9 days, the wound size remarkably reduced, indicating that WJ-MSCs seeded on an amniotic membrane could be an efficient option to improve the healing effect in chronic diabetic wounds [168]. As well, implantation of autologous biograft comprising autologous skin fibroblasts on biodegradable collagen membrane accompanied with autologous BM-MSCs resulted in reduced wound size and also promoted vascularity of the dermis in DFU patients [169]. Moreover, autologous BM-MSC can be safely and efficiently delivered to wounds using a fibrin spray system [172]. In vivo, tracking of green GFP + MSC verified presence of GFP + blood vessels, implying that the injected cells could persist and act to induce the wound repair process [172]. Another source of MSCs, BM mononuclear cells (BMMNCs), also have shown the capability to support wound healing in patients with chronic limb ischemia with acceptable safety and feasibility [173]. Other findings also proved that autologous BM-MSC therapy could be a safe and effective candidate for recurrent bullosis diabeticorum, a rare presentation of skin manifestation, as evidenced by improved clinical outcomes and averted lower limb amputation [170]. Besides, evaluation of safety and efficacy of intravenous transplantation of allogeneic human UC-MSCs in patients with recessive dystrophic epidermolysis bullosa (RDEB) outlined the potent capacity of UC-MSCs to exert safety and transient clinical benefits [171]. Indeed, assessment of the disease severity score, wound assessment, itch and pain score, and quality of life in 6 patients with RDEB revealed that injection of UC-MSCs was well tolerated, without serious adverse events. Analyses explicated that UCB-MSC infusion stimulated M2 macrophage polarization and diminished mast cell infiltration in RDEB skin [171]. Preliminary reports also have verified the safety, tolerability and modest efficacy of allogeneic BM-MSCs therapy in chronic graft-versus-host disease (cGvHD) patients with skin manifestation likely caused by MSCs immunomodulatory attributes [174, 175]. On the other hand, topical human amniotic membrane (AM)-MSCs-CM alone and with vitamins C or E was currently used for healing chronic plantar ulcers in leprosy (CPUL) [163]. Vitamin E belongs to antioxidant and anti-inflammatory attributes, and vitamin C also shows antioxidant, anti-inflammatory, and collagen synthesis properties [163]. Among all groups, the mean change in ulcer size was highest in the hAM-MSC-CM plus vitamin E group, inferring better development of wound healing. Moreover, there were no severe side effects or complications among all treated groups [163].

## Conclusion and prospect

As described, MSC’s differentiation competencies accompanied with their promising other attributes, inducing the release of anti-inflammatory and pro-angiogenic mediators, outline their significance to provoke wound healing and skin regeneration. Notwithstanding, robust inconsistency in the delivery protocols, wound models, and MSCs populations among accomplished investigations make it problematic to delineate the impacts of delivery time, delivery route, delivery systems, and also the number of injected cells on MSCs engraftment outcomes. Besides, MSCs-exosomes therapy has become a groundbreaking cell-free plan, compromising the apprehensions about the MSC's direct utility. However, the inadequate production of exosomes from parental cells hurdles their large-scale generation, which in turn, revokes their application in the clinic. Of course, it has been evidenced that culture of parental MSCs in hollow fiber three-dimensional (3D) culture system [176, 177] or MSCs seeding on biomaterial such as 45S5 Bioglass® (BG), [178, 179] or Avitene Ultrafoam collagen hemostat could support secretion marked rates of the exosomes [180]. Further, defining the reliable dependable potency tests for determining the efficacy of exosome-based therapeutics is urgently required.

## Data Availability

Not applicable.

## Abbreviations

MSCs: Mesenchymal stromal cells
TME: Tumor microenvironment
ECM: Extracellular matrix
BM: Bone marrow
UC: Umbilical cord
AT: Adipose tissue
WJ: Wharton’s jelly
miRs: MicroRNAs
TGFβ: Transforming growth factor *β*
